# Autologous transplantation of adipose-derived stromal cells combined with sevoflurane ameliorates acute lung injury induced by cecal ligation and puncture in rats

**DOI:** 10.1038/s41598-020-70767-8

**Published:** 2020-08-13

**Authors:** Zuodi Liang, Heng Zhou, Rurong Tang, Shuo Zhang, Xiaohuan Chen, Ling Pei

**Affiliations:** Anesthesiology Department, The First Hospital of China Medical University, 155 Nanjing Bei Street, Shenyang, 110001 China

**Keywords:** Stem cells, Adult stem cells, Mesenchymal stem cells, Respiration, Stem-cell research

## Abstract

Adipose-derived stromal cells (ADSCs) have excellent capacities for regeneration and tissue protection, while sevoflurane, as a requisite component of surgical procedures, has shown therapeutic benefit in animal models of sepsis. This study therefore determined if the combination of sevoflurane and ADSCs exerted additional protective effects against acute lung injury (ALI) induced by cecal ligation and puncture (CLP) in rats. The animals were randomized into five groups: (sham operation (group I), CLP followed by mechanical ventilation (group II), CLP plus sevoflurane at 0.5 minimum alveolar concentration (group III), CLP plus intravenous autologous 5 × 10^6^ ADSCs (group IV), and CLP plus sevoflurane and ADSCs (group V). Levels of the pro-inflammatory cytokines tumor necrosis factor-α, transforming growth factor-β1, interleukin-1β and interleukin-6 were significantly increased in CLP rats. Moreover, epithelial sodium channel expression levels and activities of Na/K-ATPase and alveolar fluid clearance were significantly reduced in CLP-induced ALI rats. ADSCs improved all these parameters, and these effects were further enhanced by the addition of sevoflurane. In conclusion, combined treatment with ADSCs and sevoflurane is superior to either ADSCs or sevoflurane therapy alone for preventing ALI. This beneficial effect may be partly due to improved alveolar fluid clearance by the paracrine or systemic production of keratinocyte growth factor and via anti-inflammatory properties.

## Introduction

Acute respiratory distress syndrome (ARDS) and its early stage, acute lung injury (ALI), represent a devastating clinical syndrome characterized by lung tissue edema and acute hypoxemic respiratory failure, finally leading to lung fibrogenesis. Despite extensive research efforts, mortality among critically ill patients remains high (about 40%)^1^.

Alveolar fluid clearance (AFC) is generally believed to be the main mechanism responsible for clearing edema fluid from airspaces into the lung interstitium^1^. Sodium ions enter alveolar type II epithelial cells primarily through the epithelial sodium channel (ENaC) expressed at the apical surface, composed of α, β, and γ subunits, and are pumped out by Na/K-ATPase on the basolateral surface, driving osmotic water transport^2–4^. However, AFC can be reduced by multiple pathways that impair ENaC and/or Na/K-ATPase, such as high tidal volume ventilation, hypoxia, and pro-inflammatory cytokines^5–7^. Previous studies found that patients with ALI/ARDS were also characterized by reduced AFC. Clearance of excessive pulmonary edema is thus key to ensuring effective treatment and improving survival^8,9^.

Increasing numbers of in vivo experimental and preliminary clinical studies have identified mesenchymal stem cells (MSCs) as a promising therapy for various pulmonary diseases, including ALI^10–12^. MSCs have the capacity to restore injured tissues by producing several growth factors, as well as immunomodulatory and anti-inflammatory molecules. Previous cell-based treatments have focused on bone marrow-derived stem cells (BMSCs), which have demonstrated therapeutic functions in human and rodent tissue models of ALI/ARDS^13–16^. However, there are several obstacles to the clinical application of BMSCs, including the fact that their isolation is an invasive, painful, and relatively expensive process and more importantly, the numbers of cells that can be obtained from bone marrow is low^17,18^. ADSCs have also received attention because of their positive outcomes in terms of tissue regeneration and repair. Adipose tissue includes a larger percentage (5%) of stem cells compared with bone marrow (0.01%)^19^. Furthermore, ADSCs are well suited to allograft transplantation because of a lack of major histocompatibility complex class II molecules^20^. ADSCs also have a stronger anti-inflammatory ability than BMSCs because they secrete higher levels of bioactive factors such as hepatocyte growth factor and nerve growth factor, which may account for their regeneration-enhancing properties^21^. In a recent study, ADSCs has been shown to ameliorate the ALI in rat model of CLP induced-sepsis^22^. Further research is therefore needed to determine the suitability of ADSCs for treating ALI and to investigate their possible mechanisms.

Sevoflurane is an inhaled and volatile anesthetic widely used for general anesthesia and for intensive-care sedation. In addition to its anesthetic properties, sevoflurane has been shown to attenuate pulmonary and systemic inflammation^23^, reduce alveolar edema^24^, and improve gas exchange^25^ in experimental models of lipopolysaccharide (LPS) exposure. Sevoflurane also restored ENaC function and enhanced Na/K-ATPase activity via immunomodulatory effects^26^.

However, the effects of ADSC transplantation and sevoflurane on ENaC expression and Na/K-ATPase function in ALI are largely unknown. In this study, we examined the treatment potential and mechanisms of ADSC transplantation and sevoflurane for resolving pulmonary edema, and further determined if ALI could be further improved by the combination of ADSCs and sevoflurane.

## Results

### Characterization of ADSCs

**Figure 1:**
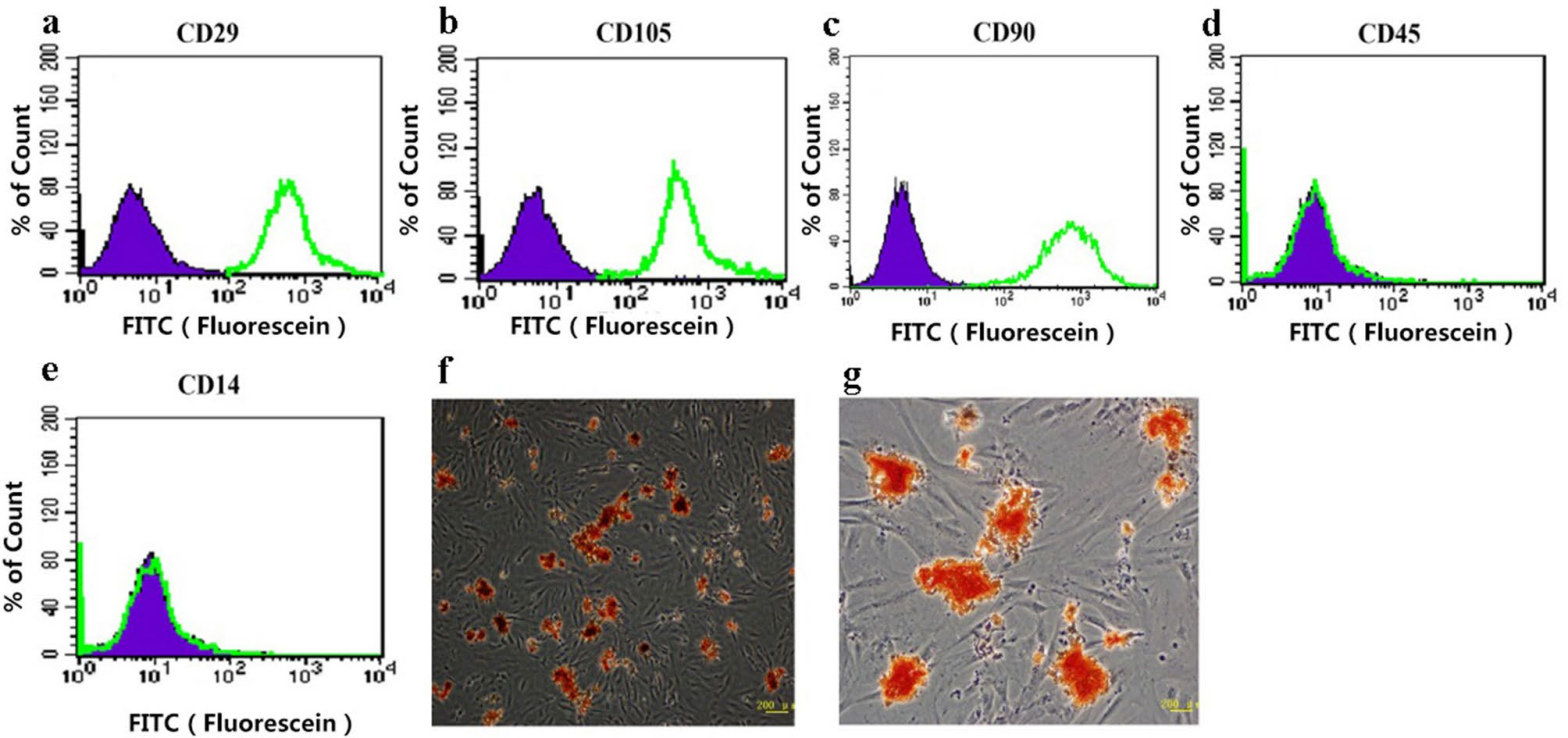
(**a–e**) Surface markers of ADSCs at passage 3 determined by flow cytometry. (**f**) Oil Red-O staining after adipogenic induction. (**g**) Alizarin Red staining after osteogenic induction. ADSCs were positive for CD29, CD90, and CD105, but negative for CD45 and CD14. ADSCs could be induced to differentiate into mature adipocytes and osteoblasts.

ADSCs isolated from subcutaneous inguinal fat pads were spindle-like adherent cells, which can differentiate into the primary mesenchymal lineages of osteocytes and adipocytes. ADSCs expressed the phenotypic markers CD90, CD105, and CD29, but not CD14 and CD45, as shown by flow cytometry (Fig. 1a–e)^27^. The ability of these cells to differentiate into osteoblasts and adipocytes was demonstrated by the deposition of calcium and intracellular lipid droplets shown by Alizarin Red and Oil Red-O staining, respectively (Fig. 1f,g). All these results showed that we were able to isolate and culture the ADSCs. We used the third-generation stem cells cultured for 2 weeks as research cells.

### ADSCs attenuated CLP-induced ALI

**Figure 2:**
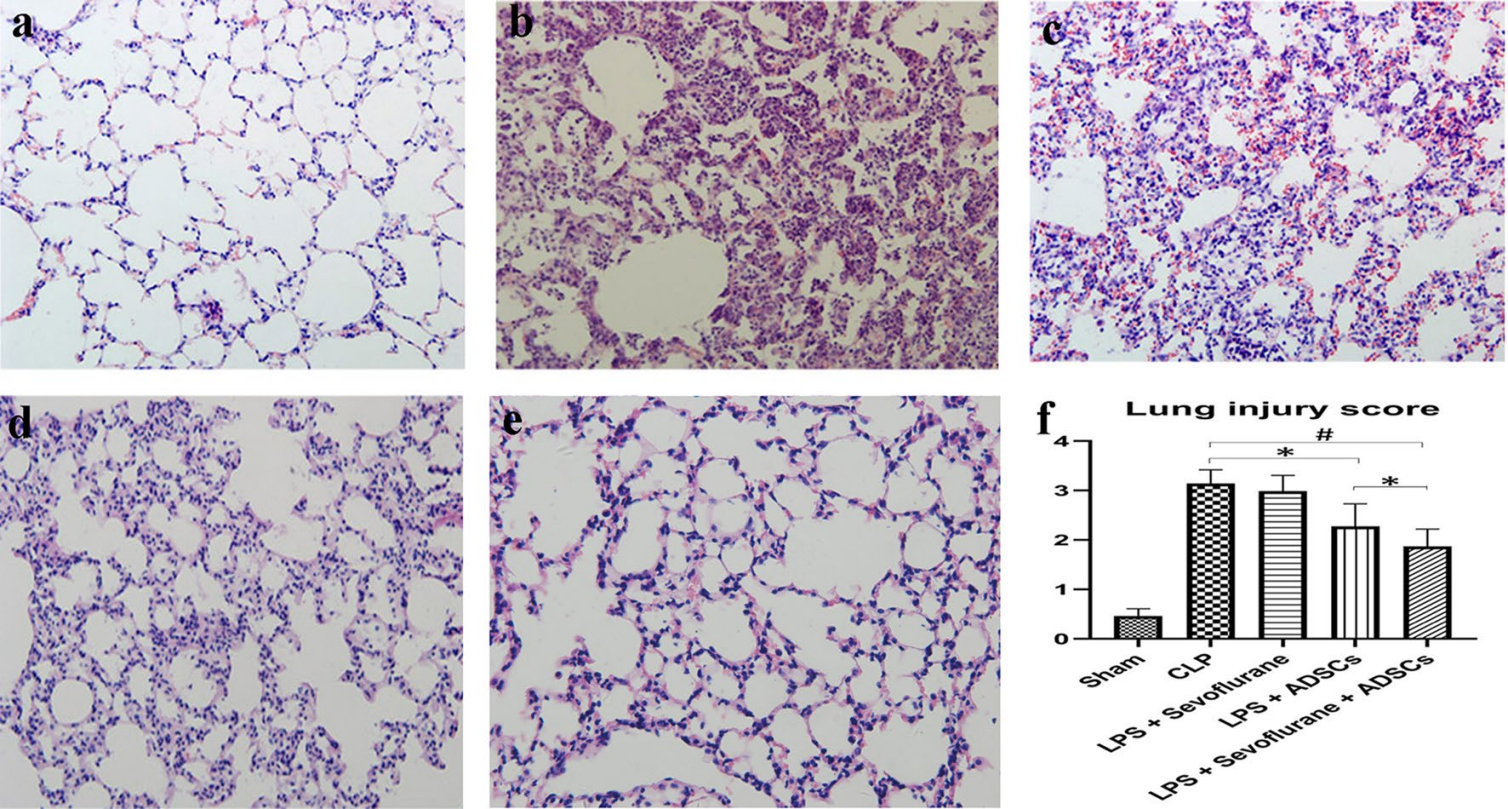
Effects of ADSCs and sevoflurane on lung histopathology. (**a**) Sham group (I); (**b**) CLP group (II); (**c**) CLP + sevoflurane group (III); (**d**) CLP + ADSC group (IV); and (**e**) CLP + sevoflurane + ADSC group (V). (**f**) Lung injury score. Normal lung tissue structures were seen in the sham group. CLP induced prominent lesions compared with control rats. These effects were little improved in the sevoflurane group, but were ameliorated by ADSCs and further by ADSCs + sevoflurane. The computerized lung injury scores were consistent with the H&E results. Data presented as mean ± standard deviation. *p < 0.05; ^#^p < 0.01; ^&^p < 0.001.

Lung tissue structure evaluated by hematoxylin and eosin (H&E) staining showed normal structures in the control group (group I; Fig. 2a). In comparison, inflammatory cell infiltration, intra-alveolar and interstitial patchy hemorrhage, thickening of the alveolar septa, and edema were observed in CLP-induced lung tissue (Group II; Fig. 2b). These changes were slightly improved by sevoflurane (group III; Fig. 2c), and were also ameliorated by ADSC treatment (group IV; Fig. 2d,e). However, the alveolar architectural and histological changes were further ameliorated by combined ADSC-sevoflurane treatment (Fig. 2e). The histological changes were reflected by similar trends in lung injury scores (p < 0.01 for group II vs group V; p < 0.05 for group II vs group IV and group IV vs group V, Fig. 2f).

### Total protein, cytokine, and chemokine levels in ALI

**Figure 3:**
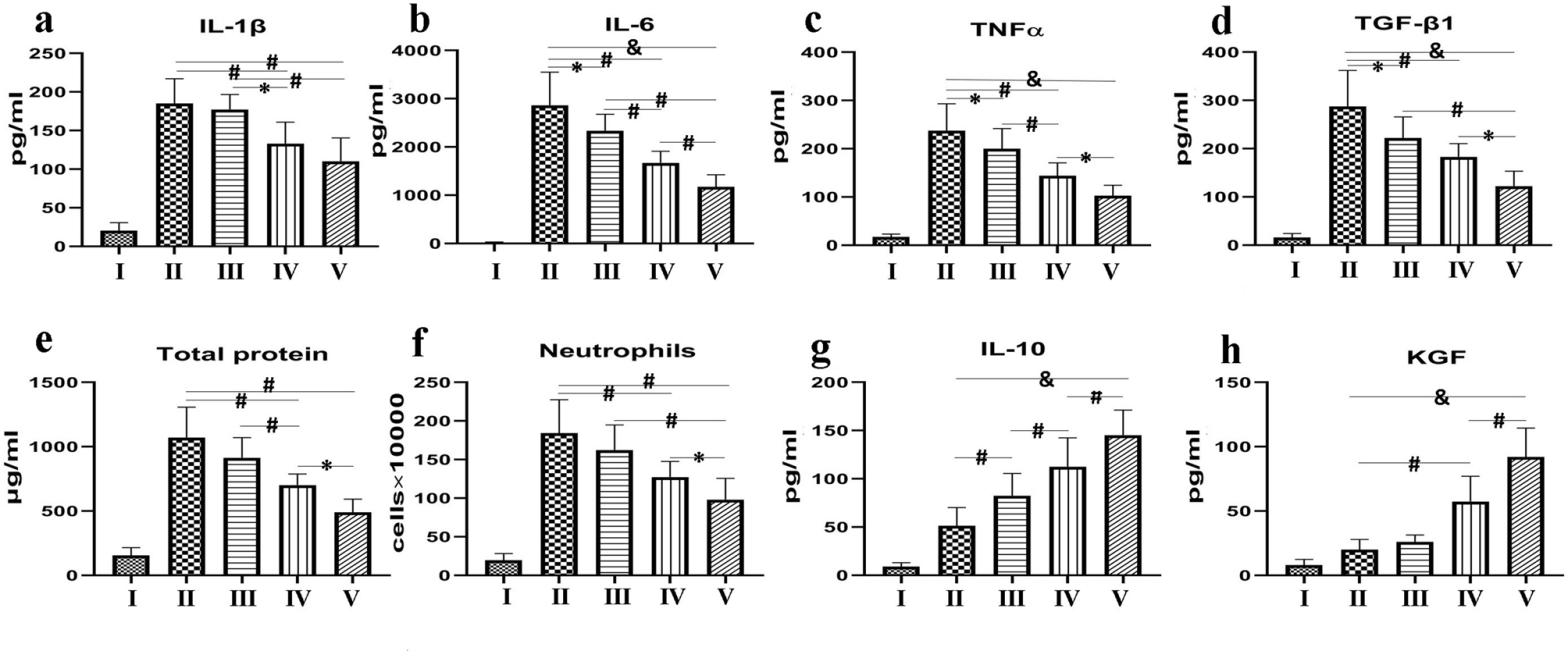
Effects of ADSCs and sevoflurane on total protein, neutrophil infiltration, and cytokine and chemokine responses in BALF. ADSCs reduced CLP-induced increases in TNF-α, IL-6, IL-1β, protein, and TGF-β1 levels and neutrophil counts. Sevoflurane also significantly reduced TNF-α, IL-6, and TGF-β1 levels. IL-10 and KGF were significantly increased in the ADSC group. These changes were increased by ADSC + sevoflurane treatment. (**a**) IL-β1; (**b**) IL-6; (**c**) TNF-α; (**d**) TGF-β1; (**e**) total protein; (**f**) neutrophil count; (**g**) IL-10; and (**h**) KGF. Data presented as mean ± standard deviation. *p < 0.05; ^#^p < 0.01; ^&^p < 0.001.

ADSC transplantation significantly alleviated CLP-induced increases in interleukin (IL)-1β, tumor necrosis factor (TNF)-α, total protein, IL-6, and transforming growth factor (TGF)-β1 levels, as well as bronchoalveolar fluid (BALF) neutrophil counts (all p < 0.01) (Fig. 3a–f). In contrast, lung levels of IL-10 (Fig. 3g), anti-inflammatory cytokines, and the epithelium-specific keratinocyte growth factor (KGF) (Fig. 3h) were significantly increased in the ADSC group. Sevoflurane significantly increased IL-10 (p < 0.01) (Fig. 3g) and ameliorated levels of IL-6, TGF-β1, and TNF-α compared with the CLP group (p < 0.05 or p < 0.01) (Fig. 3b–d). Notably, levels of inflammatory cytokines, neutrophil counts, and protein levels were further decreased in group V (combined treatment) compared with group IV (ADSCs) (p < 0.05 or p < 0.01) (Fig. 3a–f). Similarly, IL-10 and alveolar concentrations of KGF were significantly higher in the ADSC combined with sevoflurane group compared with the ADSC alone group (p < 0.05) (Fig. 3g,h).

### ADSCs increased gene and protein expression levels of ENaC in ALI

**Figure 4:**
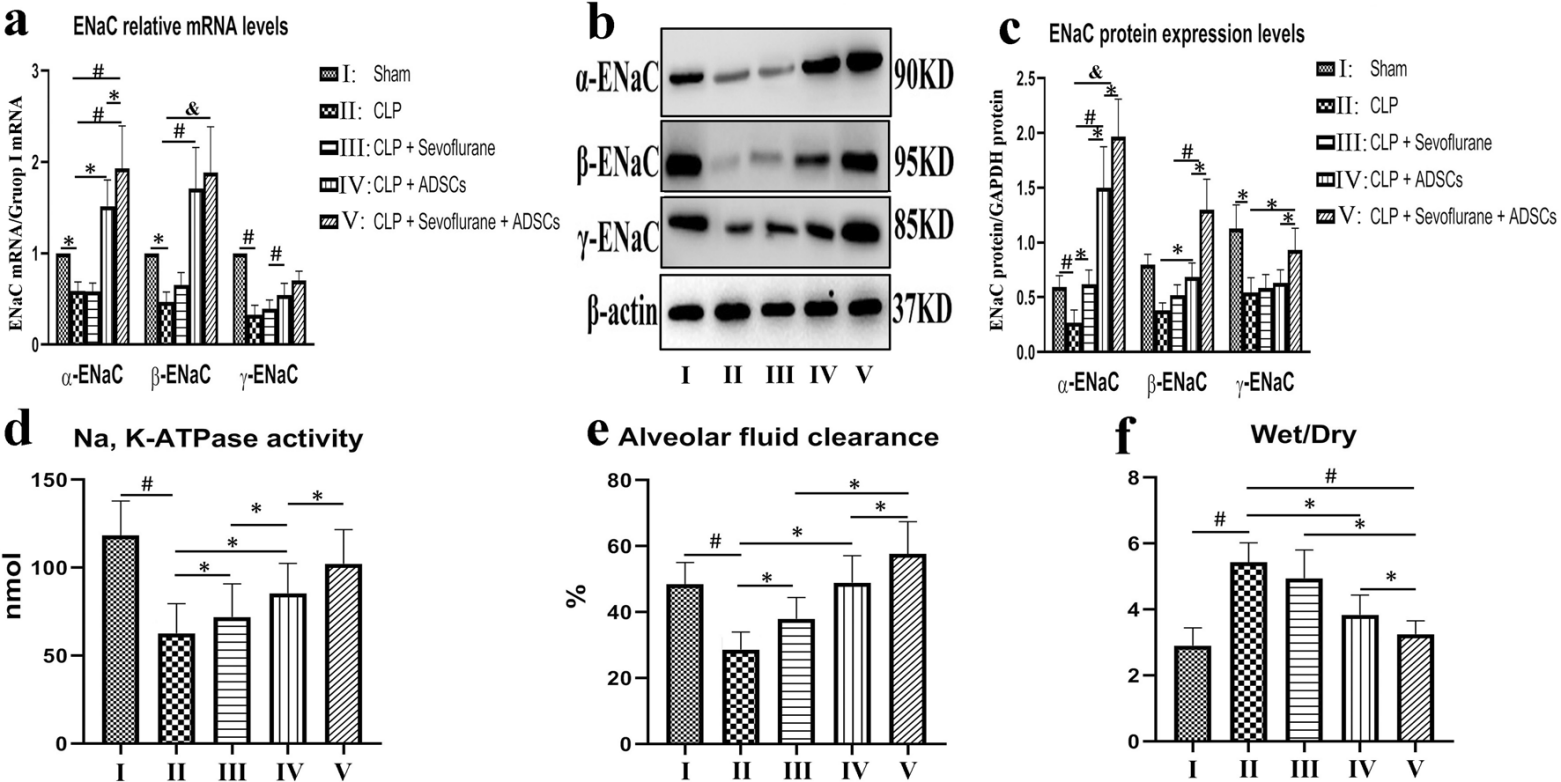
Effects of ADSCs and sevoflurane on gene (**a**) and protein (**b**,**c**) expression levels of ENaC subunits; (**d**) Na/K-ATPase activity; (**e**) AFC; and (**f**) lung W/D ratio. ADSCs increased ENaC subunit expression at the protein and mRNA levels. Na/K-ATPase pump activity and AFC were significantly improved by ADSCs combined with sevoflurane. The W/D ratio as a measure of pulmonary edema was significantly reduced in group V compared with the other groups. Data presented as mean ± standard deviation. *p < 0.05; ^#^p < 0.01; ^&^p < 0.001.

Expression levels of the three ENaC subunits were assessed by western blot analysis and polymerase chain reaction (PCR) to clarify the possible relationships between ADSCs and AFC in rat lung tissues. ENaC mRNA levels were significantly reduced in CLP compared with control rats (p < 0.01), but were significantly improved by ADSC transplantation (p < 0.01) (Fig. 4a). Levels of α-ENaC mRNA were significantly increased in groups IV and V compared with group II (p < 0.01). Sevoflurane (group III) had no significant effect on α-ENaC mRNA, but levels were significantly increased by the combination of ADSCs and sevoflurane (group V, p < 0.01) (Fig. 4a). β-ENaC mRNA levels were also significantly increased in groups IV and V compared with group II (p < 0.01), and γ-ENaC mRNA levels showed a similar trend (Fig. 4a). However, combined ADSC and sevoflurane treatment (group V) had no significant effect on β-ENaC and γ-ENaC mRNA levels compared with ADSCs alone (group IV).

α-ENaC protein expression in the apical membrane was significantly reduced by CLP (by 48%) compared with the control group (group II vs group I, p < 0.01) (Fig. 4b,c), and was restored to 156% and 183% by ADSCs without (group IV) and with sevoflurane (group V), respectively (p < 0.01) (Fig. 4b,c). The changes in β-ENaC and γ-ENaC protein levels were similar to the trend for α-ENaC (p < 0.05) (Fig. 4b,c). The addition of sevoflurane (group V) significantly increased expression levels of all three ENaC subunits compared with ADSCs alone (group IV).

### ADSCs up-regulated Na/K-ATPase activity

Na/K-ATPase activity at 12 h after CLP was reduced by approximately 47% in group II compared with group I, and was significantly restored to 68% and 90% in groups IV and V compared with group II (p < 0.01) (Fig. 4d). Moreover, sevoflurane alone significantly improved Na/K-ATPase pump activity (group III vs group II, p < 0.05) (Fig. 4d), and also significantly improved the activity compared with ADSC alone (group V vs group IV, p < 0.05) (Fig. 4d).

### ADSCs promoted AFC in ALI

We detected AFC to examine the correlation between changes in ENaC, Na/K-ATPase, and AFC. As expected, AFC was significantly reduced in the CLP group (group II) compared with the control group (group I), and treatment with ADSCs significantly enhanced AFC rates compared with group II (p < 0.01) (Fig. 4e). Moreover, AFC was significantly improved by sevoflurane alone (group III vs group II), and the addition of sevoflurane also significantly improved AFC compared with ADSCs alone (group V vs group IV, p < 0.01) (Fig. 4e).

We estimated the volume of lung water in ALI using the lung wet/dry weight (W/D) ratio. The W/D ratio was significantly increased in the CLP group (group II) compared with the control group (group I). However, the W/D ratio was significantly reduced in rats treated with ADSCs (groups IV and V) (p < 0.01) (Fig. 4f), and was significantly further reduced by the addition of sevoflurane (group V vs group IV, p < 0.05) (Fig. 4f). These results were consistent with the trends in AFC.

### Effects of ADSCs and sevoflurane on survival rate

**Figure 5:**
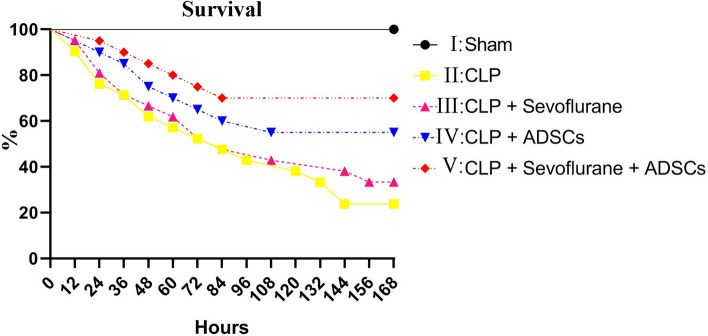
Effects of ADSCs and sevoflurane on survival. Survival rates were analyzed by log-rank tests. The survival rate at every 12 h over 7 days was significantly higher in the ADSC compared with the sevoflurane group, and survival rates at all time points were further improved in the ADSC + sevoflurane group.

The survival rate was significantly reduced (to 25%) post-injury day 7 in CLP-induced ALI rats (group II) compared with the control group (group I, 100%) (p < 0.01) (Fig. 5). This effect was ameliorated to 55% in the ADSC group (group IV) and to 70% in the ADSC plus sevoflurane group (group V) (Fig. 5). Survival was thus increased by 15% by the addition of sevoflurane compared with ADSCs alone (p < 0.05) (Fig. 5).

## Discussion

The results of the current study showed that ADSCs may have a beneficial effect in CLP-induced ALI. Moreover, the combination of ADSCs plus sevoflurane attenuated lung injury and systemic inflammation more effectively than either treatment alone, improving oxygenation and increasing survival. The beneficial effects of ADSCs were related to the inhibition of several pro-inflammatory cytokines and the production of KGF, which stimulated AFC to alleviate pulmonary edema by regulating Na/K-ATPase pump activity and increasing ENaC expression in this rat model of ALI.

CLP provides a good model of clinical sepsis^28^. In this model, peritonitis is induced by mixed intestinal flora and a cytokine response, causing ALI^29^, increased microvascular permeability, and the release of pro-inflammatory mediators including TNF-α, TGF-β1, IL-6, and IL-1β^30–32^. These in turn increase the permeability of the alveolar–capillary barrier and also influence AFC by inhibiting ENaC expression and Na/K-ATPase activity^33,34^. The current results demonstrated that rats subjected to CLP exhibited the expected pattern of ALI, including protein leakage and pulmonary edema in the alveolar spaces, significant histological lung injury, and reduced survival.

The integrity of the lung microvascular endothelium is crucial for preventing the exudation of protein-rich fluid and inflammatory cytokines from the plasma, which could further deteriorate the capacity of the lung epithelium to clear alveolar edema. The parameters detected in the present model, including lung edema, neutrophil count, histopathological lung injury score, and total protein content in BALF, were partially reversed by autologous transplantation of ADSCs. These results thus provide evidence for the amelioration of CLP-induced disruption of lung microvascular endothelial permeability and the local inflammatory response. The effects of ADSCs in improving ALI were consistent with previous studies of the therapeutic benefits of MSCs against CLP-induced injury, based on BMSCs^35,36^. The basal AFC in both alveolar epithelial type I and II cells is determined primarily by Na/K-ATPase, located on the basolateral membrane, and by ENaC, located on the apical membrane^5^. Our results indicated that ADSCs may protect AFC by increasing Na/K-ATPase activity and ENaC expression. The mechanisms underlying these effects of ADSCs may involve anti-inflammatory properties and paracrine effects. For example, α-ENaC mRNA and protein levels were reduced by TGFβ1^37^ and IL-1β^38^, and all three subunits of ENaC mRNA and protein were decreased by TNF-α^39^. In the present study, levels of the pro-inflammatory cytokines IL-1β, TGF-β1, and TNF-α were also reduced by ADSC transplantation in an ALI model. Furthermore, paracrine mechanisms, including epithelial-specific growth factors such as KGF, have been proposed to account for the effects of ADSCs in this model. The protective mechanisms of MSCs against LPS-induced lung injury have been examined in a perfused human lung model ex vivo, and intra-bronchial MSCs and MSC-conditioned medium restored AFC and reduced lung injury by releasing KGF^40^. Alveolar fluid transport was also improved by the administration of KGF in rat lung, by increasing α-ENaC gene expression^41^ and Na/K-ATPase activity^42^. α-ENaC is critical to maintaining fluid homeostasis in the lung, and the inability to clear pulmonary edema progressed to respiratory distress and death within 40 h after birth in α-ENaC-knockout mice^43^. β- and γ-subunits are also necessary for the proper activity of the channel^44,45^. The mice lacking β- and γ-gene influenced AFC in mice and induced severe dysfunction leading to neonatal death^46,47^. In our study, rats with ALI downregulates α-, β- and γ-ENaC expressions and ADSCs could reverse the ALI-induced reduction of three ENaC subunits levels.

Sevoflurane has been shown to reduce mortality via non-anesthetic properties in clinical patients^48^, and has been widely accepted to reduce the levels of inflammatory mediators (IL-1β, TNFα, and IL-6)^49^. Although ADSC treatment was more effective than sevoflurane in the current research, previous studies showed that sevoflurane had cytoprotective effects in ARDS and ALI^50^. These apparently conflicting results may be due to the current study setup or the chosen minimum alveolar concentration (MAC). However, in our study, sevoflurane also decreased concentrations of various pro-inflammatory factors, including IL-6, TNF-α, and TGF-β1, and increased the anti-inflammatory cytokine, IL-10, compared with the CLP group. These results are consistent with previous studies. In theory, the preventive effects of treatments can be improved by combining treatments with different mechanisms. We therefore speculated that sevoflurane with conventional mechanical ventilation and stem cell injections may be more efficacious in preventing ALI than either treatment alone. The results confirmed that the beneficial effects of ADSCs on ALI were significantly enhanced by the addition of sevoflurane, in terms of inhibiting inflammatory mediators and increasing anti-inflammatory cytokines and chemokines in lung tissue. Importantly, the combined therapy also increased the levels of KGF compared with ADSCs alone. α-, β- and γ-ENaC mRNA and protein expression and Na/K-ATPase activity were also further up-regulated in rats treated with the combined regimen compared with ADSCs alone. The changes in AFC were consistent with the above results, and the survival rate was ultimately improved by ADSC plus sevoflurane treatment.

This study employed a commonly used CLP-induced ALI model and ADSC transplantation; however, several limitations of the study should be discussed. First, the study did not include control groups including ADSCs in phosphate-buffered saline (PBS) sevoflurane and both approaches. However, we predict that these would have limited influence in this model. Second, although our results suggested that KGF secreted by ADSCs contributed to repair following CLP, we could not rule out possible contributions by other mediators. Lastly, the observational design of our study precluded any assessment of a cause-and-effect relationship between the mediators of injury and repair following CLP. This would require additional groups and will be a topic for future studies.

In conclusion, the current study indicates that autologous ADSC transplantation is superior to sevoflurane for restoring pulmonary fluid balance after CLP. However, the combination of ADSCs and sevoflurane is superior to either ADSCs or sevoflurane alone for preventing CLP-induced ALI, and can also increase the survival rate. The beneficial effects on AFC may be partly due to decreased release of inflammatory mediators, and/or the paracrine or systemic release of KGF and/or anti-inflammatory mediators.

## Methods

### Animals

Male Sprague–Dawley rats (7–8 weeks old, body weight 259 ± 20 g) were kept in sterile conditions. All procedures and experiments involving animals were conducted according to the ARRIVE guidelines and approved by the Animal Use and Care Council of China Medical University (approval no.: 201806). Animals were sacrificed according to the relevant ethical standards.

### Isolation, expansion, and phenotyping of ADSCs

ADSCs were separated from the subcutaneous inguinal fat pads of the rats by enzymatic digestion and cultured as described previously^51^.

### CLP-induced ALI and administration of ADSCs

ADSCs were isolated and cultured for 2 weeks. Approximately 5–10 × 10^6^ ADSCs were obtained from each rat, as reported previously^52,53^. Two weeks after surgery, sepsis was induced by CLP, as described previously^28^. Briefly, rats were anesthetized by intraperitoneal injection of xylazine (5 mg/kg) and ketamine (60 mg/kg), placed in a supine position and intubated through a 16G tracheostoma (Becton Dickinson, USA), and mechanically ventilated at a tidal volume of 8 ml/kg and respiratory rate of 100/min (Harvard Apparatus, Holliston, MA, USA). The lower abdomen was exposed by a midline incision and the cecum was identified and isolated. One third of the cecum was ligated and subjected to a single through and through puncture using an 18-gauge needle, and then gently squeezed to expel fecal material. The intestinal tract was inserted into the abdominal cavity and the abdomen was completely closed. A stable body temperature of 37 °C was maintained using a heating pad. A total of 190 rats were divided randomly into five groups (n = 18 for examinations and n = 20 for survival rate). Group I: sham group; animals were anesthetized and ventilated without CLP after closure of the abdominal wall. Group II: CLP group; rats were anesthetized and ventilated followed by CLP. Group III: CLP + sevoflurane group; rats underwent CLP for 2 h and were then exposed to 0.5 MAC (1%) sevoflurane using a sevoflurane vaporizer (VetEquip) for 10 h in 50% oxygen/air. Group IV: CLP + ADSCs group; rats underwent CLP for 2 h and autologous ADSCs (5 × 10^6^) were then slowly infused via a jugular venous cannula. Group V: CLP + sevoflurane + ADSCs group; rats underwent CLP followed by the addition of ADSCs as in Group IV and received sevoflurane as in Group III. Hydration was maintained by infusion of warmed saline (0.1 l/kg). All rats were paralyzed with continuous administration of 0.5 μg/g/h pentobarbital and muscle relaxation was achieved using 0.5 mg/kg cisatracurium once an hour. Animals were euthanized by an overdosed injection of xylazine and ketamine at 12 h after CLP.

### Obtaining and processing BALF

BALF was obtaining and proceesing as our reported previously^54^. After exsanguination, the trachea was cannulated and the right main bronchus was clamped with a hemostat. BALF was obtained from the left lung (n = 6) by flushing the lung and airways three times with 3 ml cold (4 °C) saline solution. 90% Recovered BALF was centrifuged at 3,000 rpm for 10 min at 4 °C. The supernatants were frozen at − 80 °C for subsequent analysis and the cell pellets were suspended in 100 μl PBS. The numbers of cells were counted (Beckman Coulter). The cells were stained with Wright–Giemsa stain (Sigma, USA) and the percentage of neutrophils in the BALF was determined by counting 100 cells per field.

The concentrations of IL-1β, IL-6, TGF-β1, TNF-α, IL-10, and KGF in the supernatant were determined using enzyme-linked immunosorbent assay kits, and protein levels were detected by BCA protein assay (Pierce, Rockford, IL, USA). All experiments were carried out according to the manufacturer’s instructions.

### Lung W/D weight ratio

Pulmonary edema was evaluated by the W/D ratios. The upper part of the right lung lobes was excised and weighed immediately to obtain the wet weight and the tissues were then dried in an oven at 70 °C for 48 h to obtain the dry weight.

### Lung histology

Part of the right lung lobes (n = 6) were fixed in 4% paraformaldehyde and embedded in paraffin, and 5-mm thick sections were cut and stained with H&E. Lung pathology was evaluated by light microscopy based on an assessment of interstitial infiltrates, peribronchial and perivascular inflammation, alveolar infiltrates, alveolar hemorrhage and hyaline membranes, as determined by an independent pathologist blinded to the groupings. Lung injury was scored on a 5-point scale, as described previously^55^.

### Biotinylation of apical ENaC subunits

Biotinylation of lung lobes was accomplished using a cell-surface protein purification and biotinylation kit (Pierce), as described previously^56^. Briefly, rats were killed and residual blood was removed immediately by transection of the caudal vena cava. The lung was then filled with 0.9% NaCl using a 20-gauge catheter via the pulmonary artery until white, followed by perfusion with sulfo-NHS-SS-biotin for 30 min using an 18-gauge cannula inserted into the trachea at room temperature. New biotin was instilled every 10 min after the fluid was removed. The lung tissues were then rinsed and homogenized in lysis buffer. Biotinylated proteins were isolated by mixing the protein lysate with NeutrAvidin gel followed by elution with sodium dodecyl sulfate–polyacrylamide gel electrophoresis (SDS-PAGE) sample buffer.

### RNA extraction and real-time PCR analysis

Total RNA was isolated from the lung lobe as reported previously^54^ using an RNA extraction kit (Qiagen, Hilden, Germany) in accordance with the manufacturer’s protocol. PCR comprised pre-denaturation at 95 °C for 60 s, followed by 30 cycles of 95 °C for 5 s and 60 °C for 31 s. The following primer sequences were used to amplify α-, β-, and γ-ENaC: α-ENaC, 5′-CAT GCA AGG ACT GGG GAA GG-3′ (forward) and 3′-TGG TCA TGA TCC TGC TT-5′ (reverse); β-ENaC, 5-AGA AGA AGG CCA TGT GG TTC C-3′ (forward) and 3′-GCT CAG GTA GGT CTG GAT GAA G-5′ (reverse); and γ–ENaC, 5′-AGA AGA AGG CCA TGT GGT TCC-3′(forward) and 3′-GCT CAG GTA GGT CTG GAT GAA-5′ (reverse).

Glyceraldehyde phosphate 3-dehydrogenase was chosen as the internal reference. The relative mRNA expression levels were quantified using the 2^−∆∆Ct^ method. Each experiment was repeated in triplicate.

### Western blotting analysis

Total proteins were extracted using an extraction kit (Pierce) and separated by SDS-PAGE (10%) according to the manufacturer’s instructions. The proteins were then incubated with primary antibodies to α, β, and γ-ENaC (1:300 dilution; Santa) or β-actin (1:500 dilution; Abcam) overnight at 4 °C. The membranes were then washed three times and incubated with horseradish peroxidase-conjugated secondary antibody (1:5,000 dilution, Santa) and the protein bands were visualized using chemiluminescence reagents (Pierce), according to the manufacturer’s instructions.

### Na/K-ATPase function

Basolateral membrane proteins were isolated from the peripheral lung and the hydrolytic activity of Na/K-ATPase was assessed as described previously^57^, by measuring the rate of inorganic phosphate release from ATP in the absence and presence of the selective Na/K-ATPase inhibitor, ouabain (5 mM).

### AFC in live rats

AFC was determined by the change in concentration of Evans blue-tagged albumin in an iso-osmolar alveolus instilled with or without amiloride (1 mM) and ouabain (5 × 10^4^ M)^58^. AFC was calculated according to the following equation: AFC = (1 − C0/C30), where C0 is the protein concentration of Evans Blue-labeled albumin before instillation and C30 is the albumin concentration after 30 min of ventilation.

### Survival

Survival rate was determined as described previously^59^. ALI was induced in rats as described above. ADSCs and/or sevoflurane with ventilation were administered for 12 h, starting 2 h after CLP-induced ALI. Animals were monitored for 7 days (n = 20 per group).

### Statistical analysis

All quantitative data were described as mean ± standard deviation. Statistical analysis was carried out using SPSS 22.0 software (SPSS Inc., Chicago, IL, USA). Survival data were compared by log-rank tests. Groups were compared by ANOVA, Mann–Whitney tests, post hoc Bonferroni and Dunn’s analysis, and Student–Newman–Keuls post hoc test. A p value < 0.05 was considered significant.

## Supplementary information

Supplementary Information

## Data Availability

The data that support the findings of this study are available from the corresponding author upon reasonable request.
