# Supplementary material for: Autologous transplantation of adipose-derived stromal cells combined with sevoflurane ameliorates acute lung injury induced by cecal ligation and puncture in rats
Source: Sci Rep. 2020 Aug 13;10:13760. doi: 10.1038/s41598-020-70767-8 (PMC7426944; doi:10.1038/s41598-020-70767-8)

**Autologous transplantation of adipose-derived stromal cells combined with sevoflurane ameliorates acute lung injury induced by cecal ligation and puncture in rats**

ZuoDi, Liang^1^, Heng, Zhou^1^, RuRong, Tang^1^, Shuo Zhang^1^, XiaoHuan, Chen^1^, Ling Pei^1*^**.**

^1^Anesthesiology Department, the First Hospital of China Medical University, Shenyang, China. 155 Nanjing Bei Street, Shenyang 110001, China. Tel.: +86-26-83282432.

* Correspondence

Ling Pei, E-mail: LingPei49@sina.com

Anesthesiology Department, the First Hospital of China Medical University, Shenyang, China

**Original images of western blot**

Figure 4B α-ENaC


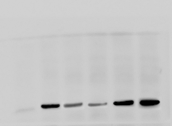


Figure 4B β-ENaC


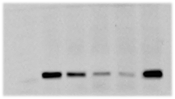


Figure 4B γ-ENaC


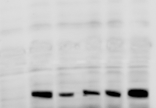


Figure 4B β-actin


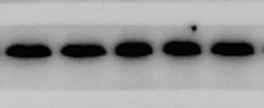

Supplement: Supplementary file 1 — Supplementary Information [file 41598_2020_70767_MOESM1_ESM.docx]
